# Biosurveillance of Selected Pathogens with Zoonotic Potential in a Zoo

**DOI:** 10.3390/pathogens10040428

**Published:** 2021-04-03

**Authors:** Pavel Kvapil, Joško Račnik, Marjan Kastelic, Jiřina Marková, Jean-Benjamin Murat, Kateřina Kobédová, Pavlina Pittermannová, Marie Budíková, Kamil Sedlák, Eva Bártová

**Affiliations:** 1 Ljubljana Zoo, 1000 Ljubljana, Slovenia; marjan.kastelic@zoo.si; 2 Department of Biology and Wildlife Diseases, Faculty of Veterinary Hygiene and Ecology, University of Veterinary and Pharmaceutical Sciences Brno, 60200 Brno, Czech Republic; jirina.markova@gmail.com (J.M.); katkakobedova@email.cz (K.K.); pittermannova.p@gmail.com (P.P.); bartovae@vfu.cz (E.B.); 3 Institute for Poultry, Birds, Small Mammals, and Reptiles, Faculty of Veterinary Medicine, University of Ljubljana, 1000 Ljubljana, Slovenia; josko.racnik@vf.uni-lj.si; 4 Tropical Neuroepidemiology Unit, University of Limoges, 87100 Limoges, France; secretariat.labo@ch-roanne.fr; 5 Biological Resource Center for Toxoplasma, National Reference Centre for Toxoplasmosis, Limoges University Hospital, 87100 Limoges, France; 6 Department of Mathematics and Statistics, Faculty of Science, Masaryk University, 60200 Brno, Czech Republic; marie.budikova@gmail.com; 7 Department of Virology and Serology, State Veterinary Institute Prague, Sídlištní 136/24, 16503 Prague 6, Czech Republic; kamil.sedlak@svupraha.cz

**Keywords:** toxoplasmosis, neosporosis, encephalitozoonosis, serology, biosurvelliance

## Abstract

Monitoring of infectious diseases is one of the most important pillars of preventive medicine in zoos. Screening for parasitic and bacterial infections is important to keep animals and equipment safe from pathogens that may pose a risk to animal and human health. Zoos usually contain many different animal species living in proximity with people and wild animals. As an epidemiological probe, 188 animals (122 mammals, 65 birds, and one reptile) from a zoo in Slovenia were examined for selected pathogens. Antibodies to *Toxoplasma gondii* and *Neospora caninum* were detected by ELISA in 38% (46/122) and 3% (4/122) of mammals, and in 0% (0/64) and 2% (1/57) of birds, respectively; the reptile (0/1) was negative. A statistically significant difference in *T. gondii* prevalence was found in Carnivora compared to Cetartiodactyla and primate antibodies to *Encephalitozoon cuniculi* were detected by IFAT in 44% (52/118) of mammals and 20% (11/56) of birds, respectively; the reptile (0/1) was negative. Herbivores had a higher chance of being infected with *E. cuniculi* compared to omnivores. Antibodies to *Chlamydia abortus* and *Coxiella burnetii* were not detected in any of the 74 tested zoo animals. The sera of 39 wild rodents found in the zoo were also examined; they were negative for all three parasites. The parasite *T. gondii* was detected by PCR in the tissue of two mute swans (*Cygnus olor*), three eastern house mice (*Mus musculus*), one yellow-necked field mouse *(Apodemus flavicollis*), and one striped field mouse (*A. agrarius*). Positive samples were genotyped by a single multiplex PCR assay using 15 microsatellite markers; one sample from a mute swan was characterized as type II. This micro-epidemiological study offers a better understanding of pathogens in zoo animals and an understanding of the role of zoos in biosurveillance.

## 1. Introduction

The role of zoos in biosurveillance has been demonstrated in the past [1]. Many different animal species usually live in a small area and in proximity to keepers, visitors, and other wildlife. In some situations, infectious pressure might overcome the immune defense of zoo animals, and pathogens might spread across various animal species. *T. gondii* and *N. caninum* are protozoal parasites with the ability to cause disease in a wide spectrum of animals [2]. Many species living in captivity (New World monkeys, lemurs, Pallas cats, marsupials, etc.) have died of clinical toxoplasmosis. There is also potential risk of exposure of children and the elderly to *T. gondii* oocysts excreted by the cats in zoos [3,4]. In contrast to *T. gondii*, *N. caninum* is not considered to be zoonotic, although there is serologic evidence of human exposure, primarily in immunocompromised people [5,6]. *N. caninum* was studied in recent years in terms of its veterinary importance, especially in canids, and economic losses in cattle due to reproductive problems [7,8]. Reproductive problems associated with *N. caninum* infection were reported also in southern white rhinoceros (*Ceratotherium simum simum*) from an Australian zoo [9]. *Encephalitozoon cuniculi* belongs to microsporidia group and is one of the three pathogenic species from the genus *Encephalitozoon*. Differentiation of microsporidiosis from other diseases in captive animals is difficult because of nonspecific clinical signs. This disease could remain unrecognized or misdiagnosed. In exotic animals, *E. cuniculi* usually does not cause any symptoms; nevertheless, some fatal occurrences have been recorded. [10,11,12,13,14]. *Chlamydia abortus* and *Coxiella burnetii* are obligate intracellular bacteria. The *Ch. abortus* is a zoonotic pathogen and causes reproductive failure, especially in sheep, goats, and cattle. This etiologic agent was also isolated in wild ruminants and zoo animals as a cause of abortions [15,16]. The *Co. burnetii* is the widespread zoonotic initiator of Q fever causing illnesses in human and livestock. Domestic and wild animals, birds, amphibians, and arthropods play the role of reservoirs of infection [17]. Although infection in livestock is mainly asymptomatic, it can lead to abortion, reproductive disorder, weak offspring, or infertility [18]. This disease was also diagnosed as a cause of abortion in zoo animals and was isolated from zoo and wildlife ungulates in Slovakia, Spain, and Portugal [19,20,21,22].

In this context, infectious diseases monitoring is an extremely important part of preventive veterinary medicine in zoos. A well-designed vaccination program and regular coprological examinations, including flotation and sedimentation techniques, are obligatory in order to provide good health care for animals in the zoo. A detailed screening plan for selected pathogens based on the current epidemiological situation is of imminent importance for the safety of animals and employees, as well as visitors. The possibility of discovering infectious threats, such as toxoplasmosis, neosporosis, chlamydiosis, and encephalitozoonosis, makes zoos suitable epidemiological stations. The aim of this study was to monitor selected pathogens at the Ljubljana Zoo in Slovenia and examine its role as a sentinel in biosurveillance.

## 2. Results

The presence of selected parasites (*T. gondii*, *N. caninum*, and *E. cuniculi*) and bacteria (*Ch. abortus* and *Co. burnetii*) was monitored in 188 zoo animals from Slovenia. Antibodies to *T. gondii*, *N. caninum*, and *E. cuniculi* were detected in 38% (46/122), 3% (4/122), and 44% (52/118) of mammals, and in 0% (0/64), 2% (1/57), and 20% (11/56) of birds, respectively (Table 1). One reptile was negative for *T. gondii* and *N. caninum* antibodies; *E. cuniculi* was not tested in this animal.

In mammals, the highest frequency of seropositives was found in Carnivora (78%), including two Eurasian lynxes (*Lynx lynx*), one Persian leopard (*Panthera pardus saxicolor)*, one Siberian tiger (*Panthera tigris altaica*), and three brown bears (*Ursus arctos*). Higher *T. gondii* seroprevalence was found in females compared to males and in omnivores compared to carnivores and herbivores, but without the statistical differences (*p* > 0.05). In Carnivora, prevalence of *T. gondii* was higher (7/9 animals; 78%) than in Cetardioadactyla (21/63 animals; 33%) and primates (2/8 animals; 25%). Antibodies to *N. caninum* were detected only in Cetartiodactyla (6%) without statistical differences (*p* > 0.05) in order, sex, and diet. The highest prevalence of *E. cuniculi* antibodies was found in five Lagomorpha (83%) and in 41 Cetartiodactyla (65%). Herbivores (63.2%, 95% CI: 52.3–74%) had a higher chance of being infected with *E. cuniculi* compared to omnivores (*p* = 0.0015, 13.3%, 95% CI: 0–30.5%). Antibodies to both *T. gondii* and *N. caninum* were found in 1.6% (2/122) of mammals (two domestic sheep (*Ovis orientalis aries*)), to *E. cuniculi* and *T. gondii* in 17% (16/95) of mammals (three from Carnivora and 13 from Cetartiodactyla), and to *E. cuniculi* and *N. caninum* in 3% (3/94) of mammals (from Cetartiodactyla). Two domestic sheep had antibodies to all three parasites. Antibodies to *Ch. abortus* and *Co. burnetii* were not found in any of 74 tested animals (61 Cetardiodactyla and 13 Diprotodontia). 

All the birds tested were negative for antibodies to *T. gondii* (0/64) and *Ch. abortus* (0/26). Antibodies to *N. caninum* were found in one animal (1/57), in a 14-year-old female blue-and-yellow macaw (*Ara ararauna*), and antibodies to *E. cuniculi* were found in 20% (11/56) of animals, in two 8- and 15-year-old male mute swans (*Cygnus olor*) and five female and four male helmeted guineafowls (*Numida meleagris*), 6 months to 5 years old. The presence of both antibodies to *N. caninum* and *E. cuniculi* was not proved. 

One reptile was negative for *T. gondii* and *N. caninum* antibodies; *E. cuniculi* was not tested in this animal.

Antibodies to *T. gondii*, *N. caninum*, *E. cuniculi*, and *Co. burnetii* were not found in any of the 39 wild rodents; antibodies to *Ch. abortus* were not examined.

The *T. gondii* DNA was detected by PCR in the tissue of seven of 21 (33%) animals that died at the zoo or were trapped during deratization. The positive tissues were livers of two mute swans and brains of three eastern house mice *(Mus musculus*), one yellow-necked field mouse *(Apodemus flavicollis*), and one striped field mouse *(A. agrarius*). The sample from a mute swan (*Cygnus olor*) was successfully genotyped and characterized as type II. The parasites *N. caninum* and *E. cuniculi* were not found by PCR in any of the tissue samples.

## 3. Discussion

The main source of parasitic infection in zoo animals is not well known. At the Ljubljana Zoo, as in other European zoos, feeding carnivores with raw meat that is potentially infected with *T. gondii* tissue cysts is a common practice. Infected felids can spread *T. gondii* oocysts in their environment and thus contaminate food, bedding, and water. In some zoo felids (*Otocolobus manul*), toxoplasmosis with a fatal outcome has been reported [23]. Carnivores can also be infected by hunting some small prey species with free access to the zoo, such as small wild rodents and birds. Another source of infection might be the presence of feral cats in the zoo, which can spread oocysts in their feces to zoo enclosures. In our study, *T. gondii* was detected by PCR in tissues of four wild rodents and in one eastern house mouse that died. The eastern house mouse came from a specific pathogen-free breeding compound; the route of infection of this animal remains unknown. One possibility is food contaminated with sporulated *T. gondii* oocysts due to improper storage of prepared pellets.

Fatal toxoplasmosis was described in six tammar wallabies (*Macropus eugenii*) in the Budapest Zoo and Botanical Garden between 2006 and 2010 [24]. Wallabies are known to be susceptible to *T. gondii* infection, and feral cats are suggested as the main source of the parasite in the zoo environment. In our study, a high prevalence of *T. gondii* was found in Camelids and Diprotodontia, which could be related to contamination of food, such as hay, or of the substrate material in their enclosures, which is mostly sand. A high risk of contamination of this substrate with the feces of feral cats, as well as difficulties in keeping stray cats outside the zoo (it is located in the city), increase the possibility of infection with *T. gondii* oocysts. New World primates are highly susceptible to clinical toxoplasmosis, and the infection is often fatal with various pathological manifestations [25]. High prevalence was previously recorded in primates such as the red-faced spider monkey (*Ateles paniscus*) and tufted capuchin (*Cebus apella*) in a zoo in Brazil, at 67% [26], and in a Barbary macaque (*M. sylvanus*), gorilla (*Gorilla gorilla*), chimpanzee (*Pan troglodytes*), and orangutan (*Pongo pygmaeus*) in the Czech Republic, at 45% [27]. In our study, *T. gondii* antibodies were found in 25% of primates (chimpanzee, *Pan troglodytes*). However, no clinical symptoms consistent with this infection have been observed. Ingestion of sporulated *T. gondii* oocysts in contaminated feed is presumed to be the main source of infection. Carefully washing vegetables and fruits as well as regularly cleaning the kitchen may reduce the risk of infection in this group of animals. Toxoplasmosis has been recorded in zoo birds; for example, in canaries and black-winged lories (*Eos cyanogenia*) [28]. In our study, antibodies to *T. gondii* were not found in any of the birds tested; however, two mute swans died and *T. gondii* was detected in their livers by PCR. These samples were genotyped and characterized as type II, which is known to be highly predominant in humans and animals in Europe and North America [29,30]. Water contaminated with *T. gondii* oocysts is one possible source of the infection in this case.

Antibodies to *N. caninum* were detected in a small number of animals. Of the mammals, only two domestic sheep and two European fallow deer were positive. The role of ruminants in the life cycle of this parasitic species is very well known and there are many studies describing the clinical signs and laboratory diagnosis of *Neospora*-positive captive and wild ruminants including those from zoos [8,31]. There were no clinical signs of *N. caninum* infection in the anamnestic history of positive ruminants from our study. In birds from Ljubljana zoo, antibodies to *N. caninum* were found in one female blue-and-yellow macaw (*Ara ararauna*). In psittacine birds that died from unrelated clinical conditions, *N. caninum* tissue cysts were demonstrated in the muscles of red-and-green macaw (*Ara chloropterus*) and blue-fronted Amazon parrots (*Amazona aestiva*) from Brazil using the imunohistochemical method [32]. The role of birds in the life cycle of *N. caninum* is still being investigated; however, the results of several experimental studies or studies on wild and captive birds suggest that they can serve as potential reservoirs of *Neospora* infections, especially as prey for wild canines, as well as a mechanical vector of *N. caninum* oocyst [8,33]. Birds can thus be an important source of infection for animals living in zoos in outdoor exhibitions, as well as wild canines (stray dogs, foxes, etc.) which can serve as potential definitive hosts of *N. caninum* in the area of the zoo.

*E. cuniculi* is a very important parasite of rabbits throughout Europe [34]. This is in accordance with results from our study because we found the highest prevalence of *E. cuniculi* antibodies in Lagomorpha (83%) in European rabbits (*Oryctolagus cuniculus*). Placentitis, premature birth, and perinatal death has been described in connection with *E. cuniculi* infection in an alpaca (*Vicugna pacos*) [35]. In our study, a high prevalence of *E. cuniculi* antibodies was found in Bovidae (81%) and Camelidae (67%). Antibodies to *E. cuniculi* were found in an alpaca (*Vicugna pacos*) and a guanaco (*Lama guanicoe*). One *E. cuniculi*-seropositive alpaca died, but the parasite was not detected by PCR in its tissue.

Clinical disease and positive isolation of *E. cuniculi* was reported in captive emperor tamarins (*Saguinus imperator*) and cotton-top tamarins (*Saguinus oedipus*), which seem to be highly susceptible to infection [10,11]. In our study, chimpanzees, lemurs, and gibbons were negative. Herbivores had a higher chance of being infected compared to carnivores, which could suggest the presence of *E. cuniculi* spores in the environment as a main source of infection. *E. cuniculi* infection in birds has no clinical importance, but some avian species could serve as reservoirs for these microsporidia [36]. In our study, antibodies to *E. cuniculi* were found in mute swans and helmeted guineafowls (*Numida meleagris*).

*Chlamydia abortus* and *Coxiella burnetii* are pathogens that are well known to cause abortions as well as various health problems in zoo animals [22]. The negative results obtained in our study may have been due to limited exposure, good preventive veterinary care, and suitable disinfection plans.

Zoos are institutions open to the public. A large variety of different animal species—usually with a high density of animals—open enclosures, the presence of stray cats and wild rodents, and close contact with people are important factors for transmission of diseases, often with anthropo-zoonotic potential, which raises public health concerns. Therefore, monitoring transmissible diseases in zoo animals and understanding their dynamics is of great importance and is an inseparable part of preventive health care in zoos.

## 4. Materials and Methods

The zoo is located on the outskirts of Ljubljana (coordinates 46°3′9.25″ N, 14°28′20.08″ E) and covers an area of 6 hectares with a large collection of exotic mammals, birds, and reptiles. Blood samples (*n* = 188) were collected from the most appropriate vein based on the animal species for 122 mammals, 65 birds, and 1 reptile in the years 2014 and 2015 (Table 1), and the serum was stored at −20 °C until assay. Tissue samples (brains or liver) of ten animals that died at the Ljubljana Zoo in 2015 were also examined. These included two mute swans (*Cygnus olor*), one black stork (*Coconia nigra*), two brown rats (*Rattus norvegicus* var. *alba*), one fat-tailed gerbil (*Pachyuromys duprasi*), one alpaca (*Vicugna pacos*), two Japanese quail (*Coturnix japonica*), and one eastern house mouse (*Mus musculus*) used as food for zoo animals. In general, samples from majority animals were collected during the preventive clinical health checks or elective surgical procedures for preventive purposes. Animals were clinically healthy with no obvious signs of any disease. Some of the samples were collected from the animals with clinical signs, where obtaining of samples was a necessary part of diagnostic procedure. In some zoo animals, the age was known, especially if they were born in the zoo. This varied across the species, from young to very old (even older than would be possible in the wild). In other animals, only a rough estimation of the age was made. Animals were not vaccinated against any of pathogens tested as these etiological agents were not part of the standard operation policy for vaccination in this zoo.

As a part of the deratization program at the Ljubljana Zoo, sera from 39 wild rodents (*n* = 39) and their tissue (brain or liver) (*n* = 11) were collected and used for assays. The rodents included 28 eastern house mice (*Mus musculus*), four brown rats (*Rattus norvegicus*), four yellow-necked mice (*Apodemus flavicollis*), two bank voles (*Clethrionomys glareolus*), and one striped field mouse (*A. agrarius*). All the samples used in this study were either blood samples collected during surgeries and preventive annual routines where blood was used for other reasons, or tissues from dead animals and rodents collected during the deratization program. For molecular analyses, the examined tissues were selected according to parasite distribution in tissues (the liver was used for detection of *E. cuniculi* DNA and the brain was used for detection of *T. gondii* and *N. caninum* DNA). An enzyme-linked immunosorbent assay (ELISA) was used to detect antibodies to *T. gondii*, *N. caninum*, *Co. burnetii*, and *Ch. abortus* with the ELISA kits ID Screen *T. gondii* Indirect Multi-Species, ID Screen *N. caninum* Indirect Multi-Species, ID Screen Q Fever Indirect Multi-Species, and ID Screen *Chlamydia abortus* Indirect Multi-Species (IDvet, Grabels, France), respectively. Samples with sample-to-positive ratio (S/P) for antibody ELISA of S/P ≥ 60% and S/P ≥ 50% were classified as positive for *Ch. abortus* and for the other infections mentioned above, respectively. Antibodies to *E. cuniculi* were determined with an indirect immunofluorescent antibody test using MegaScreen Fluoencephalitozoon (Megacor Diagnostic, Hörbranz, Austria) with *E. cuniculi* antigen. The specific conjugates were the following: anti-bovine, anti-goat, and anti-sheep IgG (VMRD, Pullman, Chicago, USA) for Bovidae; anti-camel IgG (VMRD) for Camelidae; anti-deer IgG (KPL Inc. Gaithersburg, MD, USA) for Cervidae; anti-horse IgG (VMRD) for Equidae; anti-cat IgG (Sigma Aldrich, St. Louis, MO, USA) for Felidae and Ursidae; anti-dog IgG (Sigma Aldrich) for Canidae; anti-pig IgG (Sigma Aldrich) for Suidae; anti-chicken (Sigma Aldrich) for Galiiformes; anti-duck (KPL Inc. Gaithersburg) for Anseriformes; and anti-budgerigar (KPL Inc. Gaithersburg) for Psittacidae, Accipitriformes, Ciconiiformes, Pelacaniformes, Rheiformes, Strigiformes, and Struthioniformes. Sera were diluted with phosphate-buffered saline twofold, starting with 1:50; samples with a titer ≥ 50 were marked as positive.

The DNA was isolated from the brain or liver by the QIAamp DNA Mini Kit (Qiagen, Courtaboeuf, France); in the case of *E. cuniculi*, the tissue (liver) was first homogenized using a tissue homogenizer. Detection of *T. gondii* DNA was done by PCR amplification of the TGR1E sequence [37]. The PCR mixture contained 3 µL of DNA, 20 µL of PPP master mix (Top-Bio s.r.o., Prague, Czech Republic), 0.5 µL of each primer (0.1 mM), and 16 µL of PCR-grade H_2_O. PCR was performed at the following conditions: 94 °C, 5 min; 35 cycles of 94 °C, 0.5 min; 68 °C, 40 s; and 72 °C, 40 s and 72 °C, 5 min. Genotyping of *T. gondii*-positive samples was performed with a single multiplex PCR assay with 15 microsatellite markers [38]. Detection of *N. caninum* DNA was done with PCR amplification of the Nc-5 region [39]. The PCR mixture contained 2 µL of DNA, 12.5 µL of PPP master mix (Top-Bio s.r.o), 0.1 µL of each primer (0.1 mM), and 10.4 µL of PCR-grade H_2_O. PCR was performed under the following conditions: 94 °C, 5 min; 35 cycles of 94 °C, 1 min; 63 °C, 30 s; and 72 °C, 1 min and 72 °C, 10 min. Detection of *E. cuniculi* DNA was done with PCR amplification of a small subunit of rRNA using the primers ECUNF/ECUNR [40]. The PCR mixture contained 1 µL of DNA, 12.5 µL of PPP master mix (Top-Bio s.r.o.), 1 µL of each primer, and 9.5 µL of PCR-grade H_2_O. PCR was performed at these conditions: 95 °C, 3 min; 35 cycles of 95 °C, 30 s; 60 °C, 30 s; and 72 °C, 1.5 min and 72 °C, 10 min. PCR products were analyzed on 2% agarose gel. 

The prevalence was statistically analyzed with Pearson’s chi-squared test for independence using Statistica Cz 12 [41] or with the Monte Carlo method using IBM SPSS Statistics 20. The null hypothesis that seroprevalence in mammals does not differ by orders, families, diet, and sex was tested. The differences were considered statistically significant when the *p*-value was ≤ 0.05. In the case of a statistically significant difference, the Scheffé multiple comparison method (Statistica Cz 12) was subsequently applied.

## Figures and Tables

**Table 1:** Results of serological examination (*T. gondii* and *N. caninum* tested by ELISA and *E. cuniculi* tested by IFAT) of 188 animals from the Ljubljana Zoo.

Order and Family		English Name	Latin Name	Number Tested	Diet	*T. gondii*		*N. caninum*		*E. cuniculi*	
						Positive	S/P (%)	Positive	S/P (%)	Positive	Titer
MAMMALIA				**122**		**46/122**		**4/122**		**52/118**	
Carnivora											
	Canidae	Eurasian wolf	*Canis lupus lupus*	1	C	neg.		neg.		NT	
	Felidae	Persian leopard	*Panthera pardus saxicolor*	1	C	1	100	neg.		1	50
		Euroasian lynx	*Lynx lynx*	2	C	2	89–120	neg.		1	200
		Siberian tiger	*Panthera tigris altaica*	1	C	1	112	neg.		neg.	
	Herpestidae	Suricate	*Suricata suricatta*	1	O	neg.		neg.		neg.	
	Ursidae	Brown bear	*Ursus arctos*	3	O	3	120–202	neg.		1	50
Cetardiodactyla								neg.			
	Bovidae	Alpine ibex	*Capra ibex*	19	H	4	63–211	neg.		14	50–1600
		Domestic goat	*Capra aegagrus hircus*	1	H	neg.		neg.		1	400
		Domestic sheep	*Ovis orientalis aries*	4	H	2	148–177	2	95–102	3	100–200
		Mouflon	*Ovis aries musimon*	8	H	2	54–138	neg.		8	400–800
	Camelidae	Bactrian camel	*Camelus bactrianus*	5	H	5	169–211	neg.		4	100–800
		Lama alpaca	*Lama guanicoe f. pacos*	2	H	1	161	neg.		1	100
		Lama guanako	*Lama guanicoe*	2	H	2	184–203	neg.		1	3 200
	Cervidae	European fallow deer	*Dama dama*	15	H	2	73–80	2	88–102	6	50
		Red deer	*Cervus elaphus*	5	H	3	58–78	neg.		3	50–200
	Suidae	Domestic pig	*Sus scrofa scrofa domesticus*	2	O	neg.		neg.		neg.	
Diprotodontia											
	Macropodidae	Red-necked wallaby	*Macropus rufogriseus*	24	H	14	71–116	neg.		NT	
Erinaceomorpha											
	Erinaceidae	Four-toed hedgehog	*Atelerix albiventris*	1	O	neg.		neg.		NT	
Lagomorpha											
	Leporidae	European rabbit	*Oryctolagus cuniculus*	6	H	neg.		neg.		5	400–12,800
Perissodactyla											
	Equidae	Domestic horse	*Equus ferus caballus*	4	H	neg.		neg.		2	100–200
		Donkey	*Equus asinus f. asinus*	1	H	neg.		neg.		neg.	
		Chapman´s zebra	*Equus guagga chapmanni*	2	H	neg.		neg.		1	50
		Shetland pony	*Equus ferus caballus*	2	H	neg.		neg.		neg.	
Primates											
	Hominidae	Common chimpanzee	*Pan troglodytes*	5	O	2	199	neg.		neg.	
	Hylobatidae	Yellow-cheeked gibbon	*Nomascus gabriellae*	1	O	neg.		neg.		neg.	
	Lemuridae	Black and white ruffed lemur	*Varecia variegata*	2	O	neg.		neg.		neg.	
Proboscidea											
	Elephantidae	Asian elephant	*EleHiphas maximus*	1	H	1	95	neg.		NT	
Rodentia											
	Caviidae	Capybara	*Hydrochoerus hydrochaeris*	1	H	1	87	neg.		neg.	
REPTILIA				**1**		**0/1**		**0/1**		**NT**	
Squamata											
	Agamidae	Frilled-neck lizard	*Chlamydosaurus kingii*	1	O	neg.		neg.		NT	
BIRDS				**65**		**0/64**		**1/57**		**11/56**	
Accipitriformes		Eurasian griffon	*Gyps fulvus*	2	Ca	neg.		NT		NT	
Anseriformes		Domestic goose	*Anser anser domesticus*	1	S	neg.		NT		NT	
		Mute swan	*Cygnus olor*	2	H	neg.		neg.		2	800
Ciconiiformes		Black stork	*Ciconia nigra*	5	F	neg.		neg.		NT	
Galiiformes		Helmeted guineafowl	*Numida meleagris*	19	S	neg.		neg.		9	50–100
		Indian peafowl	*Pavo cristatus*	1	S	neg.		neg.		neg.	
Pelecaniformes		Black-crowned night heron	*Nycticorax nycticorax*	13	F	neg.		NT		NT	
		Great white pelican	*Pelecanus onocrotalus*	4	F	neg.		neg.		NT	
Psittacidae		Blue-and-yellow macaw	*Ara ararauna*	2	Fr	neg.		1	91	neg.	
		Gray parrot	*Psittacus erithacus*	1	S	NT		NT		neg.	
		Red and green macaw	*Ara chloropterus*	2	Fr	neg.		neg.		neg.	
		Salmon-crested cockatoo	*Cacatua moluccensis*	2	Fr	neg.		NT		neg.	
		Sulphur-crested cockatoo	*Cacatua galerita*	1	Fr	neg.		neg.		neg.	
		Tanimbar corella	*Cacatua goffiniana*	1	Fr	neg.		NT		neg.	
		White cockatoo	*Cacatua alba*	1	Fr	neg.		neg.		neg.	
Rheiformes		Greater rhea	*Rhea americana*	1	S	neg.		neg.		neg.	
Strigiformes		Barn owl	*Tyto alba*	1	C	neg.		NT		NT	
		Eurasian eagle-owl	*Bubo Bubo*	3	C	neg.		neg.		NT	
		Ural owl	*Strix uralensis*	1	C	neg.		NT		NT	
Struthioniformes		Ostrich	*Struthio camelus*	2	O	neg.		neg.		NT	

C—carnivore, O—omnivore, H—herbivore, Ca—carcasses, S—seeds, F—fish, Fr—fruit, NT—not tested.

## Data Availability

The data that support the findings of this study are available on request from the corresponding author. The data are not publicly available due to privacy or ethical restrictions.
